# Essential Roles of PKA, iNOS, CD95/CD95L, and Terminal Caspases in Suppression of Eosinopoiesis by PGE2 and Other cAMP-Elevating Agents

**DOI:** 10.1155/2013/208705

**Published:** 2013-11-24

**Authors:** Bianca de Luca, Pedro Xavier-Elsas, Mônica Barradas, Ricardo A. Luz, Túlio Queto, Carla Jones, Maria Augusta Arruda, Thiago Mattar Cunha, Fernando Queiroz Cunha, Maria Ignez Gaspar-Elsas

**Affiliations:** ^1^Department of Pediatrics, Instituto Nacional de Saúde da Mulher, da Criança e do Adolescente Fernandes Figueira, FIOCRUZ, Avenue Rui Barbosa 716, 22250-020 Rio de Janeiro, RJ, Brazil; ^2^Department of Immunology, Instituto de Microbiologia Professor Paulo de Góes, Universidade Federal do Rio de Janeiro, CCS, Bloco I, Room I-2-066, 22205-020, Rio de Janeiro, Brazil; ^3^Farmanguinhos, FIOCRUZ, Avenue Comandante Guaranys No. 447, Jacarepaguá, 22775-903 Rio de Janeiro, RJ, Brazil; ^4^Department of Pharmacology, Faculdade de Medicina da USP, Avenue Bandeirantes 3900, Monte Alegre, 14049-900 Ribeirão Preto, SP, Brazil

## Abstract

Up- and downregulation of eosinopoiesis control pulmonary eosinophilia in human asthma. In mice, eosinopoiesis is suppressed in vitro by prostaglandin E2 (PGE2) and in vivo by diethylcarbamazine, through a proapoptotic mechanism sequentially requiring inducible NO synthase (iNOS) and the ligand for death receptor CD95 (CD95L). We examined the roles of iNOS, cAMP-mediated signaling, caspases, and CD95L/CD95 in suppression of eosinopoiesis by PGE2 and other agents signaling through cAMP. Bone-marrow collected from BALB/c mice, or from iNOS-, CD95-, or CD95L-deficient mutants (and wild-type controls), was cultured with interleukin-5 (IL-5), alone or associated with PGE2, cAMP-inducing/mimetic agents, caspase inhibitor zVAD-fmk, iNOS inhibitor aminoguanidine, or combinations thereof, and eosinopoiesis was evaluated at various times. PGE2, added up to 24 hours of culture, dose-dependently suppressed eosinopoiesis, by inducing apoptosis. This effect was (a) paralleled by induction of iNOS in eosinophils; (b) duplicated by sodium nitroprusside, isoproterenol, and cAMP-inducing/mimetic agents; (c) prevented by protein kinase A inhibition. NO was produced through iNOS by dibutyryl-cAMP-stimulated bone-marrow. Overall, PGE2 and isoproterenol shared a requirement for four effector elements (iNOS, CD95L, CD95, and terminal caspases), which together define a pathway targeted by several soluble up- and downmodulators of eosinopoiesis, including drugs, mediators of inflammation, and cytokines.

## 1. Introduction

Eosinophils, which are prominent in allergic inflammation [1], develop from bone-marrow colony-forming progenitors through lineage-committed, non-colony-forming cells (precursors) to terminally differentiated, mature granulocytes, under the influence of interleukin-5 (IL-5) [2, 3]. IL-5 is also an important mobilization, survival, and activation factor for terminally differentiated eosinophils. Nevertheless, prostaglandin E2 (PGE2), a ubiquitous inflammatory mediator, is able to override IL-5-induced survival signals [4, 5], ultimately inducing apoptosis in developing eosinophils. This regulatory effect is dependent on the inducible NO synthase isoform (iNOS), for PGE2 is ineffective in bone-marrow lacking a functional iNOS, due to either gene inactivation or pharmacological blockade. iNOS-deficient bone-marrow is nevertheless susceptible to inhibition by NO, as shown by the ability of NO-releasing chemicals to suppress eosinopoiesis, indicating that NO acts downstream from PGE2. PGE2 induces cellular markers of apoptosis (annexin V binding, TUNEL labeling, and nucleosome release). It also requires CD95 ligand (CD95L, CD158) at a second critical step, downstream from iNOS [4], to suppress eosinopoiesis. This dual requirement for iNOS and CD95L, in an ordered sequence, as well as the biochemical evidence of apoptosis, led us to propose that eosinopoiesis is regulated by PGE2 through an iNOS-CD95L-dependent proapoptotic pathway. In human asthma and experimental models of asthma, where eosinophil infiltrates are a prominent feature of the chronic pulmonary inflammation, eosinopoiesis is rapidly and selectively upregulated following airway allergen exposure [6, 7]. We have recently shown that the stimulatory effects of airway allergen exposure on bone-marrow eosinopoiesis are prevented by diethylcarbamazine, which acts in vivo through a mechanism dependent on both iNOS and CD95L [8]. In vitro, diethylcarbamazine directly suppresses eosinopoiesis in bone-marrow culture, an effect also prevented by iNOS blockade and inactivation [8]. 

Importantly, the ability of PGE2 to induce apoptosis during eosinophil development is blocked by previous exposure to dexamethasone. This shows that interference with the signaling sequence started by PGE2 is part of the modulatory effects of a widely used anti-inflammatory drug. When apoptosis is blocked by dexamethasone, a maturation-promoting activity in PGE2 is unveiled, as shown by changes in *α*4 integrin expression, cell aggregation, and cytological maturation of eosinophils in BM culture [9]. This suggests that different signaling and effector events are mobilized by the same ligand/receptor interactions, depending on the presence or absence of immunomodulators, like glucocorticoids. This added complexity further highlights the need for characterization of these events in each experimental condition. 

On the other hand, PGE2-induced suppression of eosinopoiesis is effectively blocked by cysteinyl-leukotrienes (CysLT), which are important mediators of inflammation in asthmatic lungs [5]. In IL-5-stimulated bone-marrow culture, CysLT greatly enhance eosinopoiesis [5]. CysLT further mediate the enhancing effects of eotaxin and interleukin-13, both significant players in allergic pulmonary inflammation [10]. These observations not only demonstrate that the iNOS-CD95L pathway is relevant to the pathophysiology of experimental asthma but also further highlight the need to define the precise steps which may be blocked by CysLT and cytokines which act through CysLT. 

Given the pathway's ability to transduce both negative and positive influences from various diffusible mediators and immunomodulators, we examined its relationship to other regulatory molecules. PGE2 signals through EP2 receptors, which activate adenylyl cyclase and, consequently, cAMP-dependent protein kinase (PKA) [11]. PGE2 and cAMP-elevating agents suppress colony formation by a variety of myeloid lineages, including eosinophils [12]. Adrenergic hormones/neurotransmitters, which share these signaling mechanisms with PGE2, are known physiological regulators of bone-marrow function [13]. We have therefore compared the effects of a widely used *β*-adrenergic ligand (isoproterenol) and of other cAMP-inducing/mimetic agents on eosinopoiesis with those of PGE2 and addressed the roles played by adenylyl cyclase, PKA, iNOS, NO, CD95L/CD95, and terminal caspases, in the actions of these modulators and mediators.

## 2. Methods

### 2.1. Animals

Mice of the BALB/c (both wild-type and CD95L-deficient *gld* mutants) [14] and C57BL/6 backgrounds (both wild-type and iNOS-deficient knockout mice) [15], bred at CECAL-FIOCRUZ, Rio de Janeiro, Brazil, and CD95-deficient *lpr* mutants of the C57BL/6 background [16], bred at Faculdade de Medicina da USP, Ribeirão Preto, Brazil, were used at 6–8 weeks of age, following institutionally approved (CEUA#L010/04 and CEUA#L-002/09) protocols. Where indicated, eosinophil-null mutant mice, which lack a high-affinity binding site for the GATA-1 transcription factor [17], required for eosinophil lineage commitment, and wild-type BALB/c controls were used to confirm that eosinophils were responsible for NO production. 

### 2.2. Reagents

FCS was from Hyclone (Logan, UT); culture media RPMI 1640 from RHyClone, Thermoscientific, (Waltham, MA); PGE2 (ref.14010) from Cayman Chemical Company (Ann Arbor, MI); recombinant murine (rm) IL-5 from Pharmingen (San Diego, CA), rmFlt3-Ligand (CAT# 250-31L) from Peprotech (Rocky Hill, NJ) and rmSCF (CAT# 455-MC) from R&D Systems (Minneapolis, MN); Hanks' Balanced Salt Solution, without Phenol Red (HBSS/PhR-) (ref.H6648), L-nitroarginine (ref.N5501), sodium nitroprusside (SNP) (ref.S0501), isoproterenol hydrochloride (ref.I6504), cholera toxin (ref.C8052), anti-iNOS antibody (ref.N9657), H-89 dihydrochloride hydrate (H89) (ref.B1427) selective PKA inhibitor (*K*_i_ = 48 nM), Bisindolylmaleimide II (Bis) (ref.B3056) dual PKA/PKC inhibitor (*K*_i_ = 3.0 *μ*M and 6.0 *μ*M, resp.), adenylyl cyclase inhibitor SQ 22,536 (ref.S153), 6-Isopropoxy-9-oxoxanthene-2-carboxylic acid (AH6809) (ref.A1221), selective murine EP2 antagonist [18], and 2-acetylhydrazide 10(11H)-carboxylic acid, 8-chloro-dibenz[b,f][1,4]oxazepine-10(11H)-carboxylic acid (SC19220) (ref.S3065), preferential EP1 receptor antagonist from Sigma Chemical Co. (St Louis, MO); dibutyryl cyclic AMP (ref.D0260), forskolin (ref.F6886), Z-VAD-fmk (ref.V116), dexamethasone 21-phosphate disodium salt (ref.D1159), and aminoguanidine hydrochloride, a selective iNOS inhibitor (ref.396494) from Sigma-Aldrich (St. Louis, MO); biotinylated anti-mouse IgG antibody (ref. SC-2040) from Santa Cruz Biotechnology (Santa Cruz, CA, USA.); rolipram from Sanofi (Montpellier, France); 4-amino-5-methylamino-2,7-difluorescein diacetate (DAF-FM) (ref.D23844) from Invitrogen (São Paulo, Brazil) [19]; liquid diaminobenzidine (DAB+) (ref. K3467) solution from Dako Cytomation (Dako Denmark A/S, Glostrup, Denmark).

### 2.3. Bone-Marrow Culture

 Liquid bone-marrow culture conditions were routinely used because they allow addition of agonists and inhibitors at different times, which cannot be done in clonal culture systems, where proper mixing and diffusion of substances added after plating is restricted by the semisolid media. The previously reported effects of PGE2 and cAMP-elevating agents [12] in semisolid culture are nevertheless consistent with their suppressive effects in liquid culture as detailed here (see Results Section 3.1). Eosinophilopoiesis in liquid culture was strictly dependent on IL-5 [7], and culture conditions were adequate for demonstrating both enhancing and suppressive effects [12]. BM cells were collected from both femurs of naive mice, washed, counted in a haemocytometer, seeded at 10^6^ in 1 mL of RPMI 1640 medium, 10% FCS, and rmIL-5 (1 ng/mL; optimal dose, as previously defined [7]) in 24-well clusters, and incubated at 37°C, 5% CO_2_/95% air for 7 days. Where indicated, cultures received PGE2, isoproterenol, or one of the cAMP-elevating agents (dibutyryl-cAMP, rolipram, cholera toxin, or forskolin) known to duplicate the effects of PGE2 in semisolid culture [12]. Unless otherwise indicated, each agonist was added only once, immediately after IL-5, at the beginning of the culture period. In selected experiments, PGE2 was added at various times after initiation of the culture [9]. Where indicated, cultures also received EP inhibitors (AH6809 or SC19220), iNOS inhibitor (aminoguanidine) [8], adenylyl cyclase inhibitor (SQ22536), or protein kinase A and C inhibitors (H89 or Bis) or caspase inhibitor (zVAD-fmk) [20]. In this case, inhibitors were added before both IL-5 and the agonists they were expected to antagonize. Bone-marrow cells, both before (day 0) and after (day 7) culture, were resuspended, collected, counted, cytocentrifuged, and stained for eosinophil peroxidase (EPO), a murine eosinophil lineage-specific marker present from the earliest precursors to terminally differentiated eosinophils [21]. EPO+ cells were counted at 400x under oil. Eosinophil numbers were calculated from total and differential counts. EPO+ cell counts are in excellent agreement with counts of Giemsa-stained eosinophils and with detection of CCR3+ cells by immunofluorescence [9]. For BALB/c bone-marrow cultures established for 7 days with IL-5 alone, our largest series in this study, yield was 15.81(+1.18) × 10^4^ eosinophils/mL (mean ± SEM, *n* = 29), from an initial inoculum of 10^6^ bone-marrow cells/mL. Where indicated, bone-marrow cultures were initially expanded in RPMI 1640 medium, 20% FCS, with *flt3* ligand (100 ng/mL), and stem cell factor (100 ng/mL) for 4 days, before changing the stimulus for an additional 4 days to IL-5 alone or combined with PGE2 or isoproterenol, as described by Dyer et al. [22]. 

### 2.4. Studies on iNOS Expression and NO Production

For immunocytochemical detection of iNOS, bone-marrow liquid cultures were established with IL-5, alone or in association with PGE_2_, dexamethasone (dex.),or both for 48 h, before resuspension, collection, fixation (1% paraformaldehyde), and staining of the cells. Nonspecific binding was prevented by preincubation for 1 h in PBS containing 10% FCS. The slides were washed (3x, PBS with 1% FCS) and incubated for 1 h with primary anti-iNOS antibody, diluted 1 : 100. Unbound antibody was removed by washing as above, before incubation for 1 h with secondary rat anti-mouse IgG antibody, conjugated to alkaline phosphatase, diluted 1 : 500. Unbound antibody was removed, and the reaction was developed with the Fast Red chromogen as recommended by the manufacturer. Images shown in Results Section 3.2, Figure 2 were taken with an Olympus PM-C35DX camera from an Olympus BX-50 microscope, with an Olympus UPLANAPO (Order #OB92, Spectra Services, Ontario, NY) 40x oil objective with iris (NA 1.00–0.50; WD 0.12 mm). For direct quantitation of NO generation [19], 10^6^ bone-marrow cells from iNOS-deficient C57BL/6 and the respective wild-type control mice were preincubated with DAF-FM (10 *μ*M) in a 100 *μ*L volume of HBSS/PhR-, supplemented with 100 *μ*M L-Nitroarginine, for 30 minutes at 37°C, before washing in HBSS/PhR-, supplemented with 100 *μ*M L-arginine, for 10 minutes, at 500 × *g*, and further incubation for 8 h in 2 mL of this medium in the presence of IL-5 (1 ng/mL), alone or in association with dibutyryl cAMP (10^−6^ M), aminoguanidine (10^−4^ M), or combinations of these agents. Separate control experiments evaluated eosinophil-deficient bone-marrow [17] in these conditions. Cells were collected, washed in PBS, and submitted to flow cytometry in a FACSCalibur (Becton-Dickinson) with analysis by the SUMMIT software (v4.3, Dako), with gating in the granulocyte region, defined on the basis of forward and side scatter profiles. 

### 2.5. Statistical Analysis

Data (mean ± SEM) were analyzed by factorial analysis of variance with the Tukey HSD correction, using Systat for Windows version 4 software from Systat Inc. (Evanston, IL). Significance was set at <0.05. In all panels where data are presented as % eosinophilopoiesis suppression, defined as (EPO+ cell counts in experimental cultures/EPO+ cell counts in control cultures with IL-5 alone) × 100, this mode of display was chosen for the sake of clarity, since what is shown is the ability of a *negative* agent (receptor antagonist or cyclase/kinase inhibitor) to reverse the effect of a different *negative* agent (suppressive ligand, such as PGE2 or dbcAMP), thereby evoking a *positive* response. 

## 3. Results

### 3.1. Effects of PGE2

**Figure 1:**
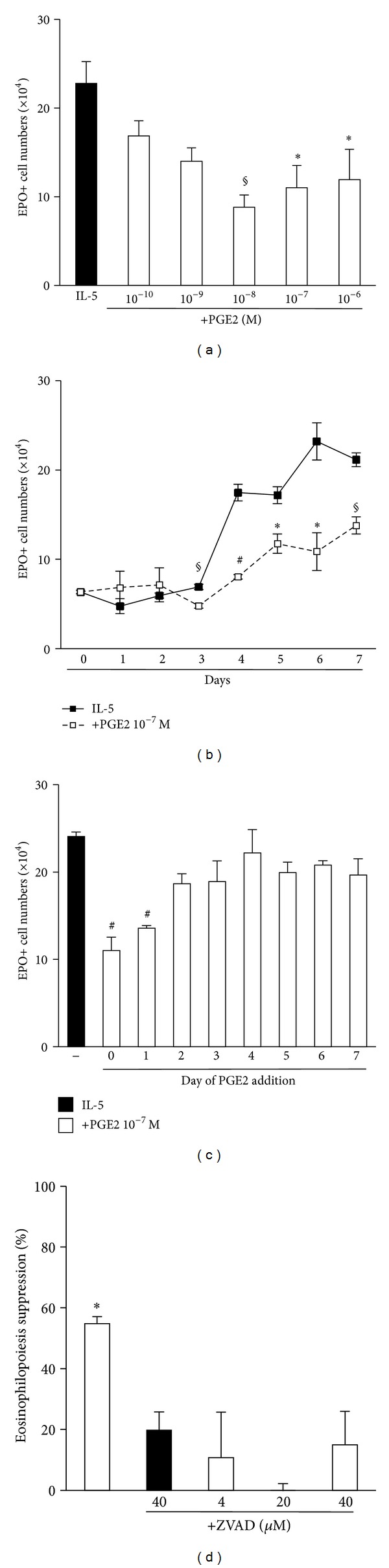
PGE2 suppresses eosinophilopoiesis by activating a caspase-dependent mechanism. Bone-marrow cultures were established in the presence of IL-5, alone or associated with PGE2, caspase inhibitor zVAD-fmk, or both. All cultures were maintained for 7 days, except for (b). All agents were added at the beginning of the culture, except for (c). Data (mean + SEM; *n* = 3) are the numbers of EPO+ cells recovered at the end of the culture ((a)–(c)) or the % eosinophilopoiesis suppression in the indicated conditions, where inhibitors were used to prevent this suppression (d). Significant differences relative to the respective IL-5 controls: *P ≤ 0.05, **P ≤ 0.01, ^§^ *P* ≤ 0.005, ^#^ *P* ≤ 0.0025.

PGE2 dose-dependently suppressed eosinophil production in BALB/c bone-marrow cultures (Figure 1(a)), as shown by the significantly decreased numbers of eosinophils recovered from 7-day cultures, established in the presence of IL-5 associated with PGE2 at 10^−6^–10^−8^ M (but not at 10^−9^–10^−10^ M), relative to the IL-5 controls. In the presence of IL-5 alone (Figure 1(b), closed squares), eosinophil numbers were significantly increased, relative to the BM inoculum (day 0), from day 4 onwards. In the presence of IL-5 and PGE2 (10^−7^ M, open squares), eosinophil recovery was also significantly increased, relative to the bone-marrow inoculums, from day 4 onwards. Nevertheless, significant differences were still observed between PGE2-treated and the respective control cultures for the entire period from day 3 to day 7, showing long-lasting suppression by PGE2. The effects of PGE2 (10^−7^ M) depended on the timing of its introduction in the culture, as significantly decreased eosinophil recovery, relative to IL-5 controls, was observed following addition at days 0-1, but not at later times (Figure 1(c)). In the absence of IL-5, PGE2 had no significant effect during a short (2 h) preincubation period, followed by media replacement and further culture in IL-5 alone for 7 days (not shown). Longer preincubation periods were not examined, as viability decreased in the absence of IL-5, even if no PGE2 is present (not shown). The long-lasting suppressive effect of PGE2 on eosinophilopoiesis depended on terminal caspases, because it was abolished by the caspase inhibitor, zVAD-fmk (4–40 *μ*M). zVAD-fmk had no effect by itself, even at the highest concentration tested (Figure 1(d)). 

Together, these observations indicate that, while PGE2 must act during the initial 24 h of culture, its suppressive effect, which involves apoptotic mechanisms, only becomes detectable 48 h later. 

### 3.2. Relationship of iNOS Expression to the Effects of PGE2 and Dexamethasone

Because PGE2 requires iNOS to suppress eosinophilopoiesis [4] and because dexamethasone prevents iNOS expression [23], we next evaluated whether iNOS expression was detectable in eosinophils and enhanced by PGE2 as well as suppressed by dexamethasone, paralleling the effects of these drugs on eosinophilopoiesis. 

**Figure 2:**
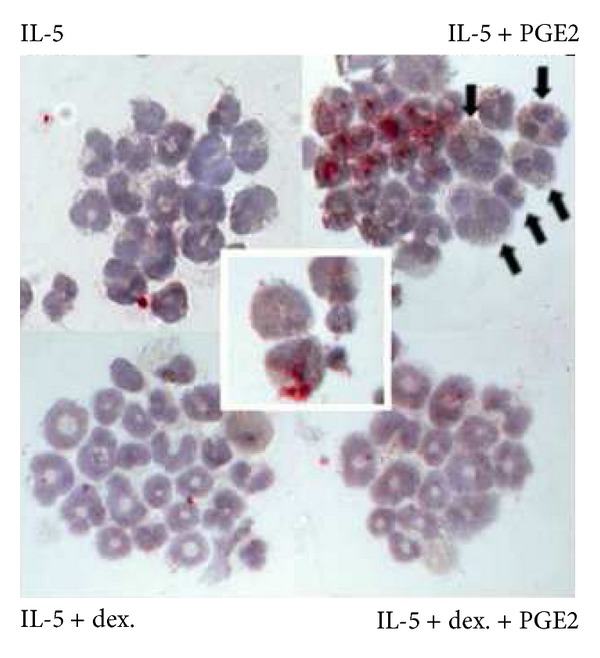
PGE2 upregulates and dexamethasone prevents iNOS expression in developing eosinophils. Bone-marrow cultures were established in the presence of IL-5, alone or associated with PGE2 (10^−7^ M), dexamethasone (10^−7^ M), or both. All cultures were maintained for 2 days, before collecting cells for detection of iNOS by immunocytochemistry. Because (bright red) cytoplasmic staining for iNOS (insert) suffers interference from (brown) cytoplasmic staining for EPO, eosinophils were identified in this experiment by their donut-shaped nucleus [6] and homotypical aggregation pattern [9], after counterstaining with Harris' hematoxylin. Arrowheads in the upper right panel point to iNOS-negative cells showing nuclear changes (chromatin condensation/nuclear fragmentation) suggestive of apoptosis (compare with upper left and lower left panels). Insert shows details of cytoplasmic staining of cells exposed to IL-5 plus PGE2. Magnification 400x (under oil).

As shown in Figure 2 (upper panel), iNOS was easily detected by immunocytochemistry in eosinophils (upper right) present at 2 days of culture in IL-5 associated with PGE2 (10^−7^ M). The strong cytoplasmic (see insert) staining of most, but not all, eosinophils present in a representative cluster strikingly contrasts with the faint staining in controls exposed to IL-5 alone (upper left). Importantly, staining was undetectable (lower left) in cultures exposed to IL-5 plus dexamethasone (10^−7^ M) and very weak in cultures exposed to both dexamethasone and PGE2 (lower right), confirming that dexamethasone prevents the upregulation of iNOS in the presence of PGE2. These patterns of iNOS expression were consistent with the detection of iNOS protein by immunoblotting in the corresponding culture conditions in separate control experiments (not shown). Both the timing of iNOS expression and its sensitivity to dexamethasone are consistent with a mechanistic relationship between upregulation of iNOS expression by PGE2 and suppression of eosinophilopoiesis. 

### 3.3. Role of Prostanoid Receptors and Caspases

**Figure 3:**
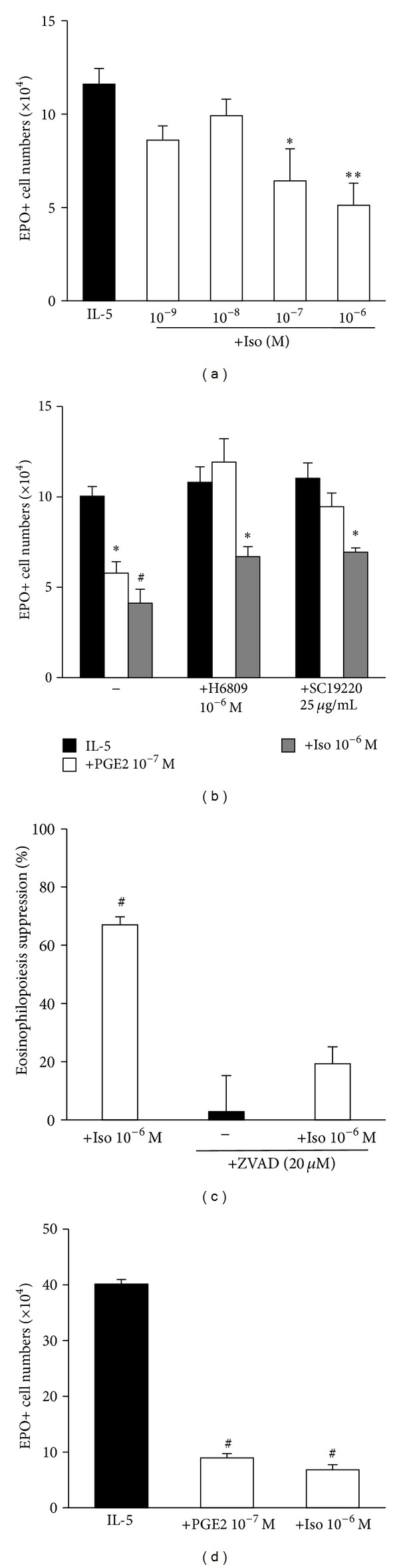
Isoproterenol duplicates the effects of PGE2 by acting at different receptors in a caspase-dependent, *flt3L*/SCF-sensitive way. (a)–(c) Bone-marrow cultures were established in the presence of IL-5, alone or associated with PGE2, isoproterenol (Iso), caspase inhibitor zVAD-fmk (ZVAD), EP2 antagonist H6809, EP4 antagonist SC19220, or the indicated combinations of these agents, which were all present from the beginning of culture for a 7-day period. (d) Bone-marrow cultures were primed with *flt3L*-SCF [22] for 4 days and further cultured for 4 days with IL-5 alone or associated with PGE2 and Iso. Data (mean + SEM; *n* = 4 in (a), *n* = 3 remaining panels) are the numbers of EPO+ cells recovered at the end of the culture ((a), (c), and (d)) or the % eosinophilopoiesis suppression in the indicated conditions, where inhibitors were used to prevent this suppression (b). Significant differences relative to the respective IL-5 controls: *P ≤ 0.05, **P ≤ 0.01, ^§^ *P* ≤ 0.005, ^#^ *P* ≤ 0.0025.

We next examined the postreceptor events that might provide a link between receptor activation by PGE2 and the induction of apoptosis at later times. We focused on cAMP-related mechanisms, because PGE2 binds to EP receptors, including two subtypes (EP2 and EP4) which primarily activate adenylyl cyclase through Gs [24]. We first evaluated whether isoproterenol, a ligand that binds to a receptor (*β*-adrenergic) unrelated to EP, would duplicate the effects of PGE2, since it mobilizes the same intracellular signaling mechanisms starting at a different surface receptor. Isoproterenol (10^−6^-10^−7^ M, but not 10^−8^-10^−9^ M) significantly suppressed eosinophilopoiesis in BALB/c bone-marrow cultures (Figure 3(a)). As previously shown for PGE2 (Figure 1(d)), the effect of isoproterenol was abolished by caspase inhibitor zVAD-fmk (Figure 3(b)), showing that terminal caspases are one effector mechanism required for the effectiveness of these two unrelated ligands. The involvement of EP receptors in the actions of PGE2 and their irrelevance to the actions of isoproterenol were confirmed by the use of the selective murine EP2 antagonist [18], AH6809, and of the EP1-selective antagonist, SC19220, which were both effective against PGE2 but ineffective against isoproterenol (Figure 3(c)). Because it has been recently reported that preexposure for 4 days to *flt3L* plus SCF primes bone-marrow for increased eosinopoiesis in the presence of IL-5 [22], we further examined the effect of priming on the suppressive activity of both ligands, comparing cultures of primed and unprimed bone-marrow cells cultured for comparable periods (Figure 3(d)). Priming by *flt3L*/SCF greatly increased eosinopoiesis, as judged from eosinophil recovery at 4 days of culture in IL-5 (compare with Figure 1(b)). However, no loss of effectiveness was found for either PGE2 (77.6 ± 4.2%) or isoproterenol (83.0 ± 6.6%), which were at least as effective after priming as in nonprimed cultures (Figures 1(d) and 3(b), resp.).

### 3.4. Role of Adenylyl Cyclase

**Figure 4:**
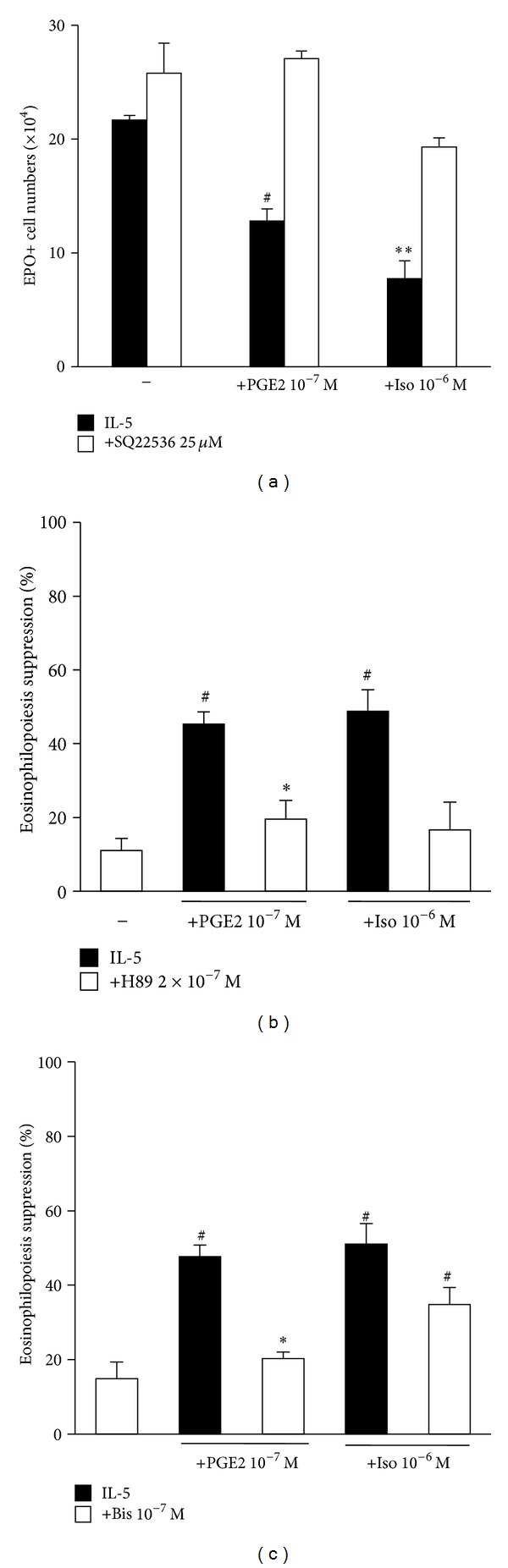
Isoproterenol and PGE2 share intracellular signaling mechanisms. Bone-marrow cultures were established in the presence of IL-5, alone or associated with adenylate cyclase inhibitor SQ22536 (a), PKA selective inhibitor H89 (b), PKC/PKA dual inhibitor Bis (c), or the indicated combinations of these agents, which were all present from the beginning of culture for a 7-day period. Data (mean + SEM; *n* = 4) are the numbers of EPO+ cells recovered at the end of the culture (a) or the % eosinophilopoiesis suppression in the indicated conditions, where inhibitors were used to prevent this suppression ((b) and (c)). Significant differences relative to the respective IL-5 controls: *P ≤ 0.05, **P ≤ 0.01, ^§^ *P* ≤ 0.005, ^#^ *P* ≤ 0.0025.

The involvement of adenylyl cyclase in transducing the signals delivered at distinct surface receptors by PGE2 and isoproterenol was confirmed by the effectiveness of SQ22536 against both agonists (Figure 4(a)). Finally, the sensitivity of the effects of both PGE2 and isoproterenol to the PKA selective inhibitor, H89 (Figure 4(b)), and the dual PKA/PKC inhibitor, Bis (Figure 4(c)), supports the hypothesis that these unrelated ligands share a requirement for PKA as a critical intracellular signaling mechanism. 

### 3.5. Effects of Receptor-Independent Agonists

To further establish the relationship of these intracellular signaling mechanisms to iNOS, we subsequently examined whether receptor-independent agonists, that either induce or mimetize increased intracellular cAMP levels, would duplicate the effects of PGE2 and isoproterenol and whether their effects would be sensitive to iNOS blockade or inactivation.

**Figure 5:**
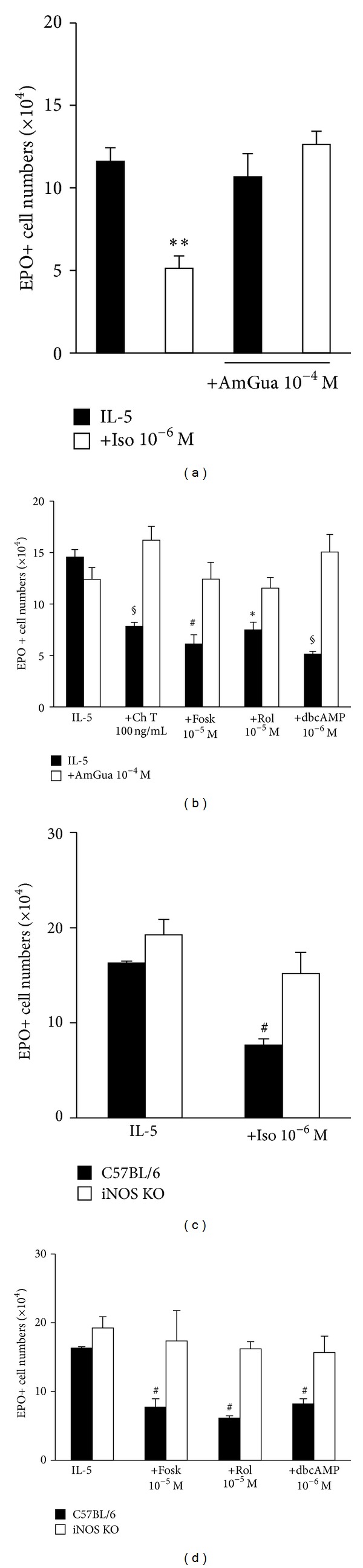
Isoproterenol and receptor-independent cAMP inducers/mimetics suppress eosinophilopoiesis through an iNOS-dependent mechanism. Bone-marrow cultures were established from BALB/c ((a) and (b)) or iNOS-deficient (iNOS KO) and their wild-type control (C57BL/6) mice ((c) and (d)), in the presence of IL-5, alone or associated with 10^−6^ M isoproterenol (Iso) ((a) and (c)) or with the indicated concentrations of cholera toxin (Ch T) (b), forskolin (Fosk), rolipram (Rol), and dibutyryl cAMP (dbcAMP) ((b) and (d)). Where indicated, iNOS inhibitor aminoguanidine (AmGua), 10^−4^ M, was used, alone or in combination with these various agonists. All agents were present from the beginning of culture for a 7-day period. Data (mean + SEM; *n* = 4 in (a), *n* = 3 remaining panels) are the numbers of EPO+ cells recovered at the end of the culture. Significant differences relative to the respective IL-5 controls: *P ≤ 0.05, **P ≤ 0.01, ^§^ *P* ≤ 0.005, ^#^ *P* ≤ 0.0025.

We initially confirmed a requirement for iNOS in the actions of isoproterenol, since these were prevented by selective iNOS inhibitor aminoguanidine, which had no significant effect in the absence of isoproterenol (Figure 5(a)). Next, we examined the effects of a wide panel of receptor-independent substances, which act to induce or to mimic elevation of intracellular cAMP levels by unrelated biochemical mechanisms. These included (a) forskolin, which directly activates adenylyl cyclase; (b) rolipram, which inhibits phosphodiesterase, leading to accumulation of its substrate, cAMP; (c) dibutyryl cAMP, which mimics the effects of cAMP and permeates cell membranes; (d) cholera toxin, which activates adenylyl cyclase by its effects on G proteins. All shared the ability to suppress eosinophilopoiesis in IL-5-stimulated bone-marrow cultures, in the absence but not in the presence of aminoguanidine (Figure 5(b)). 

For all of the agents listed above, we further confirmed the essential role for iNOS in these regulatory effects, by comparing the lack of response in iNOS-deficient bone-marrow to the responses of wild-type cells (Figures 5(c) and 5(d)). Together, these observations demonstrate the mechanistic link between cAMP signaling, iNOS function, and eosinophilopoiesis suppression.

### 3.6. Direct Evidence for NO Production in Response to cAMP Rise

**Figure 6:**
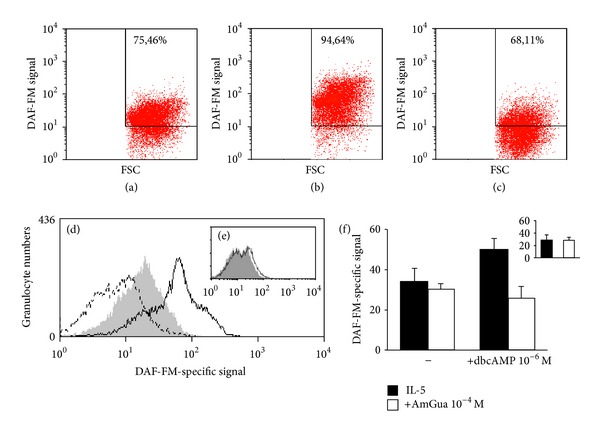
NO is generated through iNOS in response to dibutyryl cAMP in the presence of IL-5. Bone-marrow cells from wild-type BALB/c ((a)–(c)) or C57BL/6 mice ((d) and (f)) or iNOS-deficient (iNOS KO) mutant C57BL/6 mice ((e) and insert in (f)) were preincubated with DAF-FM in HBSS/PhR- containing 100 *μ*M L-arginine and further incubated for 8 h at 37°C in this medium, in the presence of IL-5, alone or associated with 10^−6^ M dibutyryl cAMP (dbcAMP), aminoguanidine (AmGua), 10^−4^ M, or combinations of these agents. Cells were harvested and incubated with DAF-FM and NO-specific fluorescence was monitored by flow cytometry with a gate in the granulocyte region. (a)–(c): dot-plot of NO production as a function of FSC for cells in the gated region, in cultures exposed to IL-5 alone (a) or associated with dbcAMP (b) or with dbcAMP plus aminoguanidine (c). Numbers indicate the % of the gated cells in the positive zone. (d)-(e): histogram of fluorescence intensities (*x*-axis) as a function of cell numbers (*y*-axis), from one representative experiment out of 3. Gray profile: IL-5 alone. Continuous line: IL-5 plus dbcAMP. Broken line: IL-5 plus dbcAMP and AmGua (wild-type controls only). (f) and insert: mean fluorescence intensities (mean + SEM; *n* = 3) from the same experiments as in (d)-(e), showing NO-specific fluorescence in wild-type control (f) and iNOS-deficient cultures (insert); (f), cultures exposed to IL-5 alone (black bar on the left) or associated with AmGua alone (white bar on the left, wild-type controls only), with dbcAMP (black bar on the right) or dbcAMP plus AmGua (white bar on the right, wild-type controls only); insert, cultures exposed to IL-5 alone (black bar) or associated with dbcAMP (white bar). (f): *P* = 0.043 for the indicated difference.

To directly confirm that NO was generated by iNOS in bone-marrow cells exposed in the presence of IL-5 to a PKA-activating stimulus, we examined by flow cytometry NO production over an 8-hour period, using an NO-sensitive probe (DAF-FM). Bone-marrow from BALB/c mice was stimulated by IL-5 (1 ng/mL), alone or in association with dibutyryl-cAMP (10^−6^ M) (Figures 6(a)–6(c)). Dibutyryl-cAMP increased the number of eosinophils emitting an NO-specific fluorescent signal (Figure 6(b)) relative to IL-5 controls (Figure 6(a)), and this increase was abolished by aminoguanidine (Figure 6(c)), confirming that the NO-specific signal was generated through iNOS. 

Next, bone-marrow from iNOS-deficient mice (Figure 6(e)) and from wild-type C57BL/6 controls (Figure 6(d)) was examined in these conditions. As expected, in iNOS-deficient bone-marrow (Figure 6(e)), dibutyryl-cAMP plus IL-5 induced no increase in NO-specific signal relative to the IL-5 controls; by contrast, the same agonistic combination increased NO-specific fluorescence relative to the IL-5 controls in wild-type bone-marrow (Figure 6(d)). This effect was blocked by aminoguanidine (Figure 6(d)). Blockade by aminoguanidine of the NO signal in wild-type bone-marrow response to dibutyryl-cAMP was statistically significant (Figure 6(f)). 

In separate control experiments with bone-marrow from mice lacking eosinophils, due to deletion of the GATA-1 high-affinity binding site required for eosinophil lineage commitment [17], no significant difference in NO-specific signals was observed between samples exposed to IL-5 plus dibutyryl-cAMP and IL-5 controls, in numbers of positive cells (*P* = 0.499) or in median fluorescence intensity (*P* = 0.408). 

### 3.7. Role of CD95

**Figure 7:**
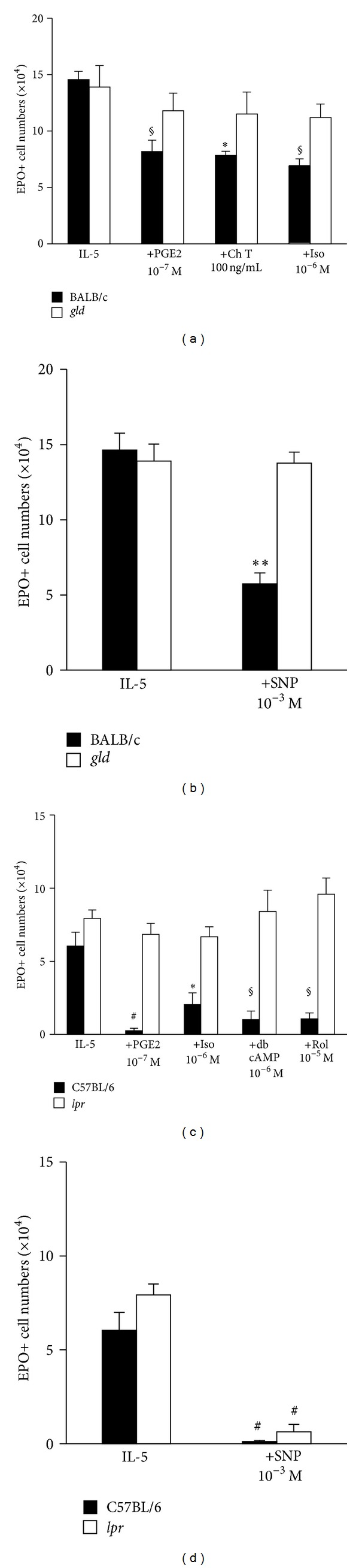
Impact of CD95L and CD95 deficiency in bone-marrow responses to cAMP-dependent agonists and to an NO donor. Bone-marrow cultures were established from BALB/c and mutant *gld* mice of the same background, lacking CD95L ((a) and (b)) or from C57BL/6 and mutant *lpr* mice of the same background, lacking CD95 ((c) and (d)), in the presence of IL-5, alone or associated with the indicated concentrations of PGE2, isoproterenol (Iso), cholera toxin (Ch T), Rolipram (Rol), and dibutyryl cAMP (dbcAMP). Where indicated, sodium nitroprusside (SNP), an NO-releasing chemical, was used to evaluate the effectiveness of NO. All agents were present from the beginning of culture for a 7-day period. Data (mean + SEM; *n* = 3 in (c), *n* = 4 remaining panels) are the numbers of EPO+ cells recovered at the end of the culture. Significant differences relative to the respective IL-5 controls: *P ≤ 0.05, **P ≤ 0.01, ^§^ *P* ≤ 0.005, ^#^ *P* ≤ 0.0025.

Finally, we addressed the issue of whether all of these different ligands converge on the same pathway to apoptosis, namely, that involving CD95L and its receptor, CD95 (Fas). CD95L-deficient bone-marrow was refractory to the suppressive effects of PGE2, cholera toxin, and isoproterenol, unlike that of wild-type BALB/c controls (Figure 7(a)). CD95-deficient bone-marrow was equally resistant to suppression by PGE2, isoproterenol, dibutyryl cAMP, and rolipram, in sharp contrast to the great sensitivity of wild-type C57BL/6 controls (Figure 7(c)). We further examined whether sodium nitroprusside (SNP), an NO donor, would be able to act on bone-marrow lacking CD95L (Figure 7(b)) and CD95 (Figure 7(d)). In agreement with previous observations made with a different NO-releasing chemical, SNAP [4], *gld* bone-marrow cells were totally resistant to SNP; by contrast, SNP retained its effectiveness in *lpr *bone-marrow cells. 

## 4. Discussion

This is, to our knowledge, the first study to establish a sequence of events strictly required for suppression of eosinopoiesis by any soluble ligand. By clearly identifying the critical steps and the experimental tools that allow their dissection, we were able to define a proapoptotic pathway which (a) begins on surface receptors; (b) requires adenylate cyclase followed by PKA signaling; (c) induces iNOS followed by iNOS-mediated NO production; and (d) ultimately triggers apoptosis through an interaction between two well-defined surface molecules, CD95L and CD95. 

As discussed below, this pathway can now be unambiguously distinguished from another sequence of events, which is initiated by the same ligand/receptor interaction but modified by glucocorticoid priming of the target cells, in a way that culminates in terminal differentiation, not in apoptotic cell death. 

Therefore, the present study demonstrates how a combination of pharmacological and genetic approaches allows one to define signals involved in cellular differentiation, as opposed to cell death, in a single hemopoietic lineage. 

Furthermore, by demonstrating that *β*-adrenergic stimuli can duplicate the effects of PGE2, this study broadens the spectrum of soluble ligands that can downregulate bone-marrow responses to allergen challenge and raises the issue of whether sympathetic fibers, abundant in bone-marrow [25], can play an attenuating or modifying role in the hematological response to allergen challenge.

The complete initial separation of pathways by ligation of distinct surface receptors by PGE2 and isoproterenol was documented by the use of the appropriate antagonists. Subsequent convergence of these pathways was demonstrated by the identical effect of blocking adenylyl cyclase on the responses to both agonists, as well as the reproduction of their effects with a wide panel of receptor-independent agonists. Sharing of both iNOS and the downstream effector molecules by these converging pathways was directly demonstrated by the inability of all the agonists tested to suppress eosinopoiesis in mice lacking iNOS, CD95L, or CD95. 

Our experiments confirmed a mechanistic link between induction and activity of NO synthase, NO generation, and CD95-dependent apoptosis. Our observations are consistent with a role for NO in inducing and activating CD95L and/or CD95, as suggested by the ability of NO donors to suppress eosinophilopoiesis in bone-marrow of wild-type but not CD95L-deficient mice [4]. 

By the second day of exposure to IL-5, the cultured eosinophils become refractory to PGE2, even though eosinophil numbers increase essentially between days 3 and 6. This suggests that the apoptosis observed at later times is the outcome of a process initiated during the initial 24 h. Since a significant impact on eosinophil numbers is first demonstrable at 72 h, the required steps should take place up to that point. Again, such an estimate is consistent with our demonstration of strong iNOS induction at the midpoint in this interval (48 h). 

We have previously demonstrated that bone-marrow cultures exposed to IL-5 in association with dexamethasone accumulate large numbers of cytologically immature eosinophils, forming aggregates, as a result of the increased expression of *α*4 integrins. In these conditions, iNOS expression is undetectable (Figure 2), and apoptosis is prevented [4]. The addition of PGE2 to these cultures downregulates *α*4 expression, decreases cellular aggregation, and allows terminal cytological differentiation, but no apoptosis occurs. These observations are entirely consistent with the evidence from this study that PGE2, in the presence of dexamethasone, did not induce iNOS expression. 

One can propose, therefore, that PGE2 induces two different sequences of events in the same target population: (a) in the *absence* of dexamethasone, it acts in the initial 24 h to start a programme involving PKA activation and inducing iNOS and NO, ultimately leading to apoptosis; (b) in the *presence* of dexamethasone, it fails to induce iNOS and NO, and apoptosis is avoided, but downmodulation of *α*4 integrins and terminal differentiation are induced. While this reinforces the notion that iNOS and NO are essential elements in the proapoptotic programme, it also allows us to predict that the maturation induced by PGE2 in dexamethasone-exposed cells is independent of iNOS. This issue will be addressed in a separate report.

A related issue which requires further investigation is whether cysteinyl-leukotrienes also affect iNOS expression, an effect which would account for their ability to enhance eosinophil survival in bone-marrow culture [5] as well as the context of asthma [26]. 

Eosinophils, which express IL-5 receptors, were also shown to express iNOS by immunocytochemistry and contribute to NO production by iNOS by flow cytometry. This is consistent with evidence from other groups [27]. The hypothesis that all critical steps take place in the bone-marrow eosinophils themselves is consistent with the high effectiveness of PGE2 and isoproterenol in *flt3L*/SCF-primed cultures, which produce functional eosinophils at high purity [22]. Although this pattern of IL-5 receptor expression and enzyme induction tends to restrict the critical events to a single lineage, NO is highly diffusible through cell membranes. Indeed, cells which do not produce NO themselves remain susceptible to the effects of NO produced by neighboring cells. In this study, iNOS expression was heterogeneous among eosinophils in the same clusters. While cells strongly expressing iNOS and producing large amounts of NO are relatively resistant to this product, since they sustain its production for extended periods, iNOS-negative cells in their neighborhood might be susceptible to NO-induced apoptosis. This would be consistent with our observation of nuclear changes suggestive of apoptosis in the neighborhood of iNOS-positive eosinophils (Figure 2). Future studies might address this issue by examining whether eosinophils expressing iNOS at high levels by day 2 are identical with, or distinct from, the cells that ultimately undergo CD95-dependent apoptosis at later times.

Interestingly, in the presence of millimolar concentrations of an NO-releasing chemical, SNP, an apparent disconnection between the impact of the mutations *lpr* and *gld* was observed in our study: while *gld* made bone-marrow resistant to the direct effect of an NO donor (Figure 7(b)), *lpr* had no impact on susceptibility to the same agent (Figure 7(d)). The refractoriness of *gld* bone-marrow strengthens the conclusion that NO is not directly cytotoxic but essentially a signaling molecule. Importantly, this signal may be more effective in the presence of IL-5, as in our study, than in its absence [28]. Disruption of this NO signal, unexpectedly, was observed with *gld* but not with *lpr*, even though *lpr* bone-marrow was as resistant to all of the previously characterized proapoptotic stimuli as *gld* bone-marrow. A possible explanation is that cells from C57BL/6-*lpr/lpr *mice, which carry an early transposable element inserted into intron 2 of the *fas *gene, retain expression of CD95 at low levels and present residual responses to CD95L+ effector cells [29–31]. Differences in the genetic background of the *gld* and *lpr* strains may also contribute to these apparent discrepancies [29]. 

While we provided direct evidence that dexamethasone blocks this pathway at one defined step, namely, the induction of iNOS, additional studies are needed, however, to define whether the downstream effector molecules, CD95 and CD95L, are also targets of dexamethasone. This possibility would be consistent with the involvement of CD95 in both apoptosis and terminal differentiation, as reported for other hemopoietic lineages: in murine erythrocytes, terminal caspases as well as CD95 played a role in inducing terminal differentiation, while sustained Raf-1 activation prevented terminal differentiation [32]; in murine bone-marrow transplantation protocols, expression and ligation of CD95 correlated with better survival and differentiation potential of donor hemopoietic cells, rather than with susceptibility to apoptosis induced by CD95L [33, 34].

## Abbreviations

PGE2: Prostaglandin E2
CD95L: Ligand for death receptor CD95
IL-5: Interleukin-5
CysLT: Cysteinyl-leukotrienes
PKA: cAMP-dependent protein kinase
EPO: Eosinophil peroxidase
ZVAD: Caspase inhibitor zVAD-fmk.
